# Exploring Symptom Overlaps: Post-COVID-19 Neurological Syndrome and Post-Concussion Syndrome in Athletes

**DOI:** 10.3390/biomedicines12071587

**Published:** 2024-07-17

**Authors:** Ioannis Mavroudis, Foivos Petridis, Antoneta Dacia Petroaie, Alin Ciobica, Fatima Zahra Kamal, Cezar Honceriu, Alin Iordache, Cătălina Ionescu, Bogdan Novac, Otilia Novac

**Affiliations:** 1 Department of Neuroscience, Leeds Teaching Hospitals NHS Trust, Leeds LS2 9JT, UK; i.mavroudis@nhs.net; 2 Faculty of Medicine, Leeds University, Leeds LS2 9JT, UK; 3 Third Department of Neurology, Aristotle University of Thessaloniki, 541 24 Thessaloniki, Greece; f_petridis83@yahoo.gr; 4 Faculty of Medicine, University of Medicine and Pharmacy “Grigore T. Popa”, University Street No. 16, 700115 Iasi, Romania; aliniordache@yahoo.com (A.I.); otilia76@gmail.com (O.N.); 5 Department of Biology, Faculty of Biology, Alexandru Ioan Cuza University of Iasi, Bd. Carol I no. 20A, 700505 Iasi, Romania; alin.ciobica@uaic.ro (A.C.); catalinaionescu81@yahoo.com (C.I.); 6 Centre of Biomedical Research, Romanian Academy, Bd. Carol I, no. 8, 700506 Iasi, Romania; 7 Academy of Romanian Scientists, Str. Splaiul Independentei no. 54, Sector 5, 050094 Bucharest, Romania; 8 “Ioan Haulica” Institute, Apollonia University, Pãcurari Street 11, 700511 Iasi, Romania; 9 Higher Institute of Nursing Professions and Health Techniques, Marrakesh 40000, Morocco; 10 Laboratory of Physical Chemistry of Processes and Materials, Faculty of Sciences and Techniques, Hassan First University, B.P. 539, Settat 26000, Morocco; 11 Faculty of Physical Education, Alexandru Ioan Cuza University of Iasi, Bd. Carol I no. 20A, 700505 Iasi, Romania; chonceri@yahoo.fr; 12 Clinical Department, Apollonia University, Păcurari Street 11, 700511 Iasi, Romania

**Keywords:** COVID-19, post-concussion syndrome, athletes, neurological syndrome, sports medicine, rehabilitation

## Abstract

The COVID-19 pandemic has introduced new challenges in managing neurological conditions, particularly among athletes. This paper explores the intersection of post-COVID-19 neurological syndrome (PCNS/PASC) and post-concussion syndrome (PCS), focusing on their implications in sports medicine. Our analysis covers the symptomatology, pathophysiology, and management strategies for PCNS/PASC and PPCS, with special attention paid to the unique challenges faced by athletes recovering from these conditions, including the risk of symptom exacerbation and prolonged recovery. Key findings reveal that both PCNS/PASC and PPCS present with overlapping symptoms such as cognitive difficulties, exercise intolerance, and mental health issues, but differ in specific manifestations like anosmia and ageusia, unique to COVID-19. Pathophysiological analysis reveals similarities in blood–brain barrier disruption (BBB) but differences in the extent of immune activation. Management strategies emphasize a gradual increase in physical activity, close symptom monitoring, and psychological support, with a tailored approach for athletes. Specific interventions include progressive aerobic exercises, resistance training, and cognitive rehabilitation. Furthermore, our study highlights the importance of integrating neurology, psychiatry, physical therapy, and sports medicine to develop comprehensive care strategies. Our findings underscore the dual challenge of COVID-19 and concussion in athletes, necessitating a nuanced, interdisciplinary approach to effective management. Future research should focus on the long-term neurological effects of both conditions and optimizing treatment protocols to improve patient outcomes. This comprehensive understanding is crucial for advancing the management of athletes affected by these overlapping conditions and ensuring their safe return to sports.

## 1. Introduction

In the wake of the global COVID-19 pandemic, the medical community has faced unprecedented challenges, not least among them being the emerging complexities associated with long-term sequelae of the infection. Among these, the neurological implications of COVID-19, particularly in comparison and contrast to post-concussion syndrome (PCS), have garnered significant attention [1,2]. 

For three decades, sports medicine practitioners have concentrated on the epidemic of sport-related concussions (SRC) [3]. A sport-related concussion is a common injury in sports, affecting athletes of all ages and occurring with varying incidence rates across contact and limited-contact sports [4,5]. It is prevalent among children and adolescents involved in sports [6], high school student-athletes [7], and professional athletes [8]. The advent of COVID-19 has redirected this focus to a novel intersection; athletes are now at risk of experiencing both COVID-19 and concussion, presenting a unique clinical challenge. Early in the pandemic, attention was drawn to COVID-19 cardiomyopathy and myocarditis in athletes. Recent research has focused on the cardiac implications of COVID-19 in athletes. While early concerns were raised about the increased prevalence of cardiac injury in COVID-19 patients [9,10], subsequent large-scale studies have shown a low prevalence of myocarditis in athletes recovered from COVID-19 [11]. However, the potential for cardiac complications, including myocarditis, remains a significant concern for athletes returning to play after COVID-19 infection [9,10,12]. Myocarditis is a known cause of sudden cardiac death in athletes, prompting the need for careful cardiac evaluation and risk stratification [9,11,12]. Recommendations for return-to-play protocols have been proposed, including cardiac testing such as electrocardiograms, echocardiograms, and in some cases, cardiac MRI [9,10,12]. While the clinical significance of subclinical myocarditis detected by cardiac MRI remains uncertain, ongoing research aims to determine the prevalence, severity, and clinical relevance of COVID-19-associated cardiac pathology in athletes [10,11,12].

SARS-CoV-2 has also been found to profoundly affect the central nervous system (CNS), leading to various neurological complications. These include meningitis, encephalitis, stroke, and neuromuscular disorders [13,14]. The virus may affect the CNS through direct invasion, though rare, or more commonly through the secondary effects of systemic hyper-inflammation, which can disrupt the blood–brain barrier and activate CNS immune pathways [14]. Long-term neuropsychiatric symptoms, known as Long Covid, can persist for months after infection, even in young people with mild initial disease [15]. The pathophysiological mechanisms are not fully understood but likely involve immune dysfunction and neuroinflammation. The ACE-2 receptor, used by SARS-CoV-2 to enter cells, is present in nervous system tissue, potentially explaining the virus’s neurotropism [16]. This revelation has prompted a need to understand how COVID-19 intersects with the pathophysiological processes and clinical presentations commonly seen in concussion and PCS [2]. Specifically, we aim to investigate whether COVID-19 exacerbates existing pathophysiological processes, prolongs recovery times, or introduces unique clinical challenges that complicate management strategies for athletes recovering from concussion and PCS.

The term post-COVID-19 neurological syndrome (PCNS) has been coined to describe the constellation of prolonged neurological symptoms observed in COVID-19 “long haulers” [17]. This syndrome shows notable parallels to persistent post-concussive symptoms (PPCS), which last beyond the typical recovery period following a concussion [18]. Both conditions manifest with a range of symptoms, including but not limited to, headache, cognitive difficulties, mood disturbances, sleep irregularities, and exercise intolerance. While anosmia and ageusia are more characteristic of COVID-19, the overlap in many other symptoms raises important considerations for diagnosis and treatment [17,18].

Moreover, the shared etiology of symptoms such as fatigue, brain fog, sleep disruption, and exercise intolerance in PPCS and PCNS, attributable to systemic inflammation and autonomic dysfunction, underscores the need for a nuanced approach in management. These insights are particularly pivotal in sports medicine, where athletes may be concurrently managing the repercussions of a concussion and COVID-19.

Here we aim to explore the intersection of COVID-19-related neurological syndromes and PCS, delving into their symptomatology, pathophysiology, and management strategies, with a special focus on implications for sports medicine. This paper seeks to provide a comprehensive overview of the current understanding of PCNS and PCS, emphasizing the clinical, diagnostic, and therapeutic parallels and distinctions. Through this exploration, we aim to contribute to the evolving landscape of best practices for managing these complex syndromes, particularly in sports medicine and beyond.

## 2. Pathophysiology and Symptomatology

The neurological consequences of SARS-CoV-2 have become a pivotal aspect of its overall pathogenicity, warranting a deeper exploration into its mechanisms. Central to the neurological impact of SARS-CoV-2 is the angiotensin-converting enzyme-2 (ACE2) receptors. These receptors are a key facilitator for the virus’s entry into cells, a process that notably includes neuronal cells and other cells within the brain [19]. As a result, ACE2 receptors play a crucial role in the neurological manifestations of COVID-19.

Interestingly, in milder cases of COVID-19, direct viral DNA is often not detected in brain pathology or cerebrospinal fluid. This observation suggests that the neurological symptoms may not always result directly from the viral presence in neural tissues. Instead, these symptoms are primarily attributed to the body’s immune response to the virus. Immune activation and inflammation become significant factors in this context. The immune response, while crucial for fighting the virus, can inadvertently lead to adverse neurological outcomes.

One such adverse outcome is endothelial dysfunction. The endothelium, a layer of cells lining blood vessels, plays a critical role in maintaining vascular health and function. In COVID-19, the inflammation and immune response can impair endothelial function. This impairment can lead to disruptions in the blood–brain barrier, a critical structure that regulates the passage of substances between the bloodstream and the brain [20,21,22]. The disruption of the blood–brain barrier is particularly significant because it can lead to a range of neurological complications, from mild symptoms like headaches to more severe outcomes such as encephalopathy.

Moreover, there is a noteworthy parallel between the mechanisms involved in COVID-19-related neurological impact and those in concussion pathology. In both scenarios, the disruption of the blood–brain barrier and the ensuing endothelial dysfunction play a pivotal role. This similarity suggests that there could be common therapeutic targets or strategies that could be effective in mitigating the neurological impact of both conditions. Numerous studies explore the melatonin potential as a therapeutic agent for neurological conditions, particularly PCS and COVID-19-related neurological symptoms. Barlow et al. (2020) [23] conducted a randomized clinical trial on children with PCS, finding no significant effect of melatonin compared to placebo in reducing symptoms. However, an earlier protocol by Barlow et al. (2014) [24] highlighted melatonin’s neuroprotective properties and potential benefits for PCS treatment. Wongchitrat et al. (2021) [25] reviewed melatonin’s antiviral, antioxidant, and neuroprotective properties, suggesting its potential role in treating virus-induced neuropathogenesis, including COVID-19-related neurological symptoms. Iyer et al. (2020) [26] investigated the neural correlates of melatonin treatment in pediatric PCS patients, observing changes in functional connectivity and gray matter volume associated with improved sleep parameters, despite no significant effect on overall symptom recovery.

While the direct presence of SARS-CoV-2 in neural tissues is not always evident in milder COVID-19 cases, the neurological symptoms observed are likely a consequence of systemic immune activation and inflammation. These responses, while part of the body’s defense mechanism against the virus, can inadvertently compromise the integrity of the blood–brain barrier and endothelial function, leading to a spectrum of neurological manifestations. The parallels with concussion pathology further underscore the significance of these mechanisms and may provide avenues for future research and therapeutic interventions.

### 2.1. Post-Concussion Syndrome

The pathophysiology of post-concussion syndrome (PCS) presents a complex challenge, primarily due to the intricate pathophysiological mechanisms triggered by mild traumatic brain injuries (MTBIs) or concussions [18,27,28]. 

The initial impact leads to a simultaneous series of pathophysiological changes, including neuronal depolarization, alterations in glucose metabolism, the release of excitatory neurotransmitters, changes in cerebral blood flow, and axonal dysfunction. These processes are intricately linked to the development of symptoms, cognitive impairments, and changes in motor control [28].

At the cellular level, these changes are marked by ionic shifts, notably the efflux of potassium and the influx of sodium and calcium into neurons. These ionic changes activate voltage- or ligand-gated ion channels, culminating in widespread neuronal depression, which primes cells for barrier dysfunction and hampers their ability to clear debris and resolve inflammation (Table 1) [29,30]. The subsequent attempt to restore ionic balance depletes energy reserves, leading to an energy crisis due to both high demand for ATP by ionic pumps and impaired energy delivery [29,31]. This energy mismatch, exacerbated by mitochondrial dysfunction from increased intracellular calcium, can persist for days in animal models and contributes to behavioral impairments, though its duration in humans remains uncertain [29,31].

Concussions also disrupt neuronal architecture, damaging axons and microtubules through calcium influx, phosphorylation, or mechanical stretch, and affecting cellular transportation and causing axonal swelling [29,32,33]. This vulnerability is particularly pronounced in the developing brains of children due to ongoing myelination, with advanced neuroimaging techniques like diffusion tensor imaging (DTI) offering insights into such structural disruptions [34,35].

Neuroinflammation is another critical component of concussion pathophysiology, with rodent models showing upregulation of inflammatory genes and increased microglia/macrophage presence [36,37,38,39]. Myo-inositol levels rise following concussion, indicating astrocyte activation and inflammation [40,41,42,43]. Magnetic resonance spectroscopy (MRS) has been employed to detect these subtle neuroinflammatory changes and other pathophysiological alterations in TBI cases [43,44,45,46,47,48].

The neurometabolic cascade following concussion is further complicated by the release of excitatory neurotransmitters like glutamate, altering ion channels and NMDA receptor function [29]. This leads to elevated levels of damage-associated molecular patterns (DAMPs), glutamate, and ions, which disrupt cell function [49,50,51]. ATP and HMGB1, types of DAMPs, recruit immune cells for tissue repair, while activated microglia respond to injury, associated with poor outcomes [52,53,54,55,56,57,58,59,60]. The excessive release of glutamate and the consequent overstimulation of its receptors exacerbate neuronal death through excitotoxicity, highlighting the critical role of glutamate transporters in mitigating cellular toxicity [61,62,63,64,65,66].

Research suggests that not only calcium but also sodium dysregulation contributes to cellular damage post-concussion, necessitating further investigation into sodium’s role in neurological trauma [67,68]. Persistent alterations in sodium levels post-recovery may increase the risk of adverse outcomes like memory decline [69].

### 2.2. From Pathophysiology to Symptomatology

The translation of these pathophysiological processes into the clinical presentation of PCS is a complex interplay of biological, psychological, and social factors. The hallmark symptoms of PCS—including cognitive deficits, headaches, dizziness, and emotional lability—are likely reflections of the underlying neuronal and metabolic disturbances. For instance, cognitive impairments could be rooted in disrupted neural networks and altered neurotransmitter dynamics, while headaches might originate from dysregulated cerebral blood flow and neuronal excitability.

In PCS, the persistence and interaction of these symptoms often result in a self-perpetuating cycle, where the presence of one symptom exacerbates the others, creating a complex clinical picture that challenges both diagnosis and management.

### 2.3. Symptoms of PCS

In PCS, the persistent symptoms that follow the initial injury are akin to uninvited guests who linger long after the incident, disrupting the delicate equilibrium of daily life. The clinical presentation of PCS is diverse and complex, encompassing a spectrum of symptoms that, while individually familiar, collectively form a challenging puzzle for both patients and clinicians [70].

Headaches, often described as relentless and unyielding, emerge as a prominent symptom in the aftermath of concussion. These are not ordinary headaches; they can be debilitating, disrupting the rhythm of daily activities and significantly impairing quality of life. They vary in intensity and character, sometimes throbbing incessantly, at other times presenting as a dull, constant ache that serves as a continual reminder of the injury [71].

Dizziness, another frequent companion of PCS, adds a disorienting layer to the patient’s experience. It is a sensation akin to being on an unsteady boat, where the world seems to sway unpredictably. This symptom, in its persistence, can profoundly affect balance and coordination, instilling a sense of vulnerability and uncertainty in navigating physical spaces [18,70].

Fatigue, too, is a common and particularly insidious symptom. It is not simply a feeling of tiredness but an overwhelming sense of exhaustion that can envelop patients, draining their energy and motivation. This fatigue is disproportionate to the activities undertaken and can be a significant barrier to recovery, limiting the ability to engage in both physical and cognitive rehabilitation exercises [70,72].

Cognitive difficulties, often described as brain fog, encompass a range of deficits in memory, attention, and executive function. Patients describe a frustrating sense of mental cloudiness, an inability to focus or process information as efficiently as before. This cognitive impairment can affect professional and academic pursuits, straining one’s ability to work or study effectively [18,73,74,75].

Sleep disturbances, ranging from insomnia to hypersomnia, further complicate the recovery landscape. The restorative power of sleep, so crucial in healing, becomes elusive for many. These disturbances can form a vicious cycle, where the lack of quality sleep exacerbates other symptoms, such as cognitive difficulties and mood changes [76].

Mood changes, including depression and anxiety, often weave their way into the tapestry of PCS. The chronic nature of symptoms, coupled with the disruption to daily life and activities, can lead to frustration, sadness, and worry. The psychological impact of PCS is profound, affecting not just the patients but also their support systems and relationships [73,76].

The constellation of these symptoms presents a multifaceted challenge in managing PCS. The chronicity and variability of symptoms necessitate a personalized and adaptive approach to treatment.

## 3. Comparative Analysis

### 3.1. Overlapping Symptoms

The shared spectrum of symptoms between PCNS/PASC and PCS, particularly cognitive difficulties, mood disturbances, sleep irregularities, and headaches, presents a fascinating yet perplexing clinical scenario. These symptoms, often subtle and non-specific, are akin to shadows in the vast landscape of neurological conditions—elusive, often morphing, and sometimes mirroring other neurologic disorders (Table 1).

Cognitive Difficulties: In both PCNS/PASC and PCS, patients frequently report a troubling cloudiness of thought, a phenomenon colloquially referred to as ‘brain fog’. This cognitive mist represents a significant overlap, blurring the lines between these two conditions. Patients describe challenges in concentration, memory lapses, and an overarching sense of mental sluggishness. These cognitive impairments, while not entirely unique to either condition, reflect a disruption in the intricate neural networks, possibly stemming from different pathophysiological origins [74,75].

Mood Disturbances: The labyrinth of mood disturbances in both PCNS/PASC and PCS adds another layer of complexity. Patients often traverse a spectrum ranging from irritability to more profound experiences of depression and anxiety (Table 1) [76,77]. This shared emotional landscape raises intriguing questions about the underlying neurobiological mechanisms. It suggests a potential commonality in the disruption of the neural circuits and neurotransmitter systems involved in mood regulation, albeit triggered by different etiological factors in PCNS/PASC and PCS.

Sleep Irregularities: The interplay between sleep and neurological health is profoundly exhibited in both conditions. Patients commonly report a disruption in sleep patterns, including difficulties in falling asleep, staying asleep, or experiencing restorative sleep (Table 1) [78,79,80]. This intersection of sleep disturbance provides a compelling area for further exploration, considering the critical role of sleep in neurological repair and emotional regulation.

Headaches: The prevalence of headaches in both PCNS/PASC and PCS marks a symptomatic crossroad. While the headache types may vary, their presence is a testament to the shared symptomatology (Table 1). In PCS, headaches might be attributed to the direct impact of traumatic brain injury, whereas in PCNS/PASC, the etiology could be more diverse, including inflammatory responses or vascular changes [81,82]. Another common symptom in PCS and PCNS/PASC is the presence of anosmia (loss of smell) and ageusia (loss of taste) in PCNS/PASC, symptoms that have emerged as somewhat pathognomonic of COVID-19. In post-concussion syndrome, anosmia and ageusia may result from direct trauma to the brain, particularly affecting the areas that process smell and taste sensations. These symptoms can occur immediately after the injury or develop over time. The duration and severity of anosmia and ageusia in PCS vary, depending on factors like the severity of the initial injury and individual recovery processes [83].

In the context of Long COVID, anosmia and ageusia are also common symptoms. They are thought to be related to the virus’s impact on the nervous system, especially the nerve cells involved in smell and taste (Table 1) [84]. Unlike PCS, where the cause is usually a direct physical injury to the brain, in Long COVID these symptoms might arise from the virus’s indirect effects on neural pathways.

The convergence of these symptoms in PCNS/PASC and PCS not only complicates clinical diagnosis but also presents a therapeutic conundrum. Clinicians are often tasked with deciphering these symptoms’ origins, akin to navigating a complex maze without a clear starting point. The overlapping nature of these symptoms necessitates a nuanced approach to diagnosis, one that considers a comprehensive clinical history, a careful neurological examination, and, when appropriate, ancillary testing.

Furthermore, this symptom overlap underscores the importance of a tailored, patient-centric approach in management. It challenges healthcare providers to look beyond the conventional diagnostic categories and consider a more holistic view of the patient’s symptomatology. Consequently, treatment regimens must be as dynamic and multifaceted as the symptoms, often requiring a combination of pharmacological, rehabilitative, and psychological strategies.

### 3.2. Differences in Symptomatology and Pathophysiology

In the quest to unravel the complexities of post-COVID-19 neurological syndrome (PCNS/PASC) and PCS, it becomes imperative to discern the nuanced differences that exist in their symptomatology and underlying pathophysiological mechanisms. While both conditions exhibit a convergence of symptoms, particularly in cognitive and neuropsychiatric domains, they diverge significantly in certain clinical manifestations and the intricacies of their pathogenesis [71,72,73,74,75,76,77,78,79,80,81,82,83,84].

The pathophysiological landscape of PCNS/PASC and PCS, while sharing overlapping territories, diverges in significant ways. Both conditions implicate the disruption of the blood–brain barrier (BBB), a critical structure maintaining CNS homeostasis (Table 1) [84]. The mechanisms leading to this disruption appear to diverge. In COVID-19, the BBB disruption is hypothesized to stem from a systemic inflammatory response, a byproduct of the body’s immune reaction to the viral infection. This inflammation, characterized by cytokine release and immune cell activation, has been implicated in a range of neurological symptoms observed in PCNS/PASC [85]. Conversely, in PCS, the BBB disruption is more frequently attributed to the mechanical forces exerted during the initial injury [84]. These forces result in a cascade of neuronal and vascular changes, including ionic fluxes and metabolic disturbances, which culminate in a constellation of symptoms that define PCS. Although inflammation plays a role in PCS, its nature and extent differ from the systemic immune response observed in COVID-19. The inflammatory response in PCS tends to be more localized and secondary to the primary mechanical insult.

Additionally, the specific neurological pathways affected in each condition reveal further divergences. PCNS/PASC demonstrates a peculiar involvement of neuronal populations associated with olfaction and taste, as well as potential impacts on autonomic regulation (Table 1) [82,86]. In PCS, the affected pathways often relate more to cognitive processing, vestibular function, and sometimes post-traumatic headache syndromes, reflecting the diffuse and multifocal nature of the initial mechanical injury [80,87,88,89].

These differences carry significant implications for clinical management and rehabilitation strategies. Understanding the unique pathophysiological underpinnings of each condition is crucial for developing targeted therapeutic approaches. Furthermore, these distinctions underscore the need for precision in diagnostic processes. The overlapping symptomatology can pose diagnostic challenges, particularly in cases where patients present with both conditions, such as athletes who have experienced concussions and later contract COVID-19. In such scenarios, a keen understanding of these nuances becomes paramount in disentangling the symptomatology attributable to each condition, thereby guiding effective treatment strategies.

While PCNS/PASC and PCS share common ground in their clinical presentations, the divergences in their symptomatology and pathophysiological mechanisms highlight the complexity and heterogeneity of neurological conditions. A deeper exploration into these differences not only enriches understanding but also paves the way for more tailored and effective clinical interventions.

## 4. Management and Rehabilitation Strategies

### 4.1. COVID-19 Neurological Syndrome

Current Treatment Approaches: The management of post-COVID-19 neurological syndrome (PCNS) primarily revolves around symptomatic relief and supportive care. Given the novelty of the condition, treatment protocols are continually evolving. Current strategies include cognitive rehabilitation for addressing memory and concentration issues, psychological support for mental health symptoms, and physical therapy for addressing fatigue and muscle weakness [89]. Pharmacological interventions may also be used for specific symptoms like headache or neuropathic pain [89,90].

Role of Exercise and Rehabilitation: Rehabilitation for PCNS also emphasizes the role of exercise, particularly progressive aerobic exercises and resistance training (Table 2). This approach aligns with the growing recognition of the benefits of physical activity in improving cognitive and psychological outcomes in neurological conditions (Table 1) [89]. Exercise routines are tailored to individual capabilities and are aimed at gradually improving endurance and strength without exacerbating symptoms.

### 4.2. Post-Concussion Syndrome

Treatment Modalities for PCS: The management of PCS is multifaceted, targeting specific symptoms and their underlying causes. This includes vestibular therapy for balance and dizziness, cognitive therapy for memory and concentration issues, and psychotherapy for addressing mental health challenges (Table 1). Pharmacological treatments may be used for persistent headaches, sleep disturbances, and mood disorders [80,91].

Rehabilitation Focus: Rehabilitation in PCS often involves a gradual return-to-activity protocol, commonly known as the “stepwise approach”. This protocol ensures that patients progressively increase their activity levels without triggering a resurgence of symptoms. The focus is on creating a balance between rest and activity, avoiding both physical and cognitive overexertion (Table 2) [91].

## 5. Implications for Athletes

The intersection of COVID-19 and concussion within the athletic community underscores a multifaceted challenge, requiring careful consideration of the implications for athletes’ health and their safe return to sport. This dual concern introduces a complex clinical landscape that demands a nuanced understanding and strategic approach, particularly in light of the compounded challenges presented by both conditions and the unique demands of athletic performance. 

Supporting Data: Challenges Faced by Athletes

Exercise Intolerance: Numerous studies have examined the COVID-19 impact on exercise capacity and cardiopulmonary function in athletes and physically active adults. While Komici et al. (2021) [92] found no significant reduction in exercise capacity during early recovery, other studies reported persistent limitations. Singh et al. (2021) [93] observed markedly reduced peak exercise aerobic capacity and impaired systemic oxygen extraction in recovered patients. Baratto et al. (2021) [94] noted reduced exercise capacity and augmented exercise hyperventilation, primarily due to peripheral factors like anemia and reduced oxygen extraction. Ladlow et al. (2022) [95] found that hospitalized patients and those with persistent symptoms had lower oxygen uptake at peak exercise compared to controls, even after 5 months. However, community-managed individuals who felt recovered showed no significant differences from controls. Collectively, these findings suggest that COVID-19 can have lasting effects on exercise capacity, particularly in more severe cases, highlighting the need for ongoing monitoring and rehabilitation in affected individuals. 

Cognitive Difficulties: Recent studies have highlighted the COVID-19 cognitive impacts, with evidence of persistent brain fog affecting memory, attention, and executive function. A study by Becker et al. [96] found that cognitive impairment could last seven months or longer, particularly in hospitalized patients (D’arrigo, 2022). Arbula et al. (2024) [97] reported significant attention deficits and mild executive function impairments in post-COVID patients, along with psychological alterations. Warren et al. (2022) [98] discussed the post-infectious neurocognitive syndrome associated with SARS-CoV-2, emphasizing the need for lifestyle changes and rehabilitation strategies. A comprehensive review by Daroische et al. (2021) [99] found that 15–80% of patients experienced global cognitive impairment, with specific deficits in attention, executive functions, memory, and verbal fluency. These findings underscore the cognitive assessment importance for COVID-19 patients, regardless of disease severity or treatment methods, and highlight the need for further research with larger sample sizes and control groups.

Mental Health Issues: The COVID-19 pandemic significantly impacted college students’ mental health, particularly among student-athletes. Multiple studies reported increased stress, anxiety, and depression among students during this period [100,101]. A survey of NCAA athletes revealed a rise in mental health issues, exacerbated by disrupted training schedules and competition uncertainties [102]. Female athletes were found to be more affected, reporting lower satisfaction with mental health and physical preparedness compared to male counterparts [103]. Factors contributing to mental health challenges included concerns about personal and loved ones’ health, academic performance, and decreased social interactions [100,101]. Interestingly, while the general population showed increased substance use, NCAA athletes reported no significant changes in this regard [103].

Research indicates that athletes’ brains exhibit distinct structural and functional adaptations compared to inactive athletes. Master athletes demonstrate superior cognitive function compared to sedentary counterparts, exhibiting better verbal memory and reaction times [104]. They also show larger regional brain volumes and enhanced cognitive performance [105]. Regular exercise appears to preserve brain function and increase physical health, potentially mitigating age-related cognitive decline [104].The relationship between exercise intensity and cognitive performance follows an inverted-U curve for non-athletes, while athletes maintain improved attentional behavior, even at higher exercise intensities [106]. Conversely, physical inactivity may lead to structural changes in the neurons associated with cardiovascular regulation, potentially increasing the risk of cardiovascular disease [107]. These findings collectively emphasize the importance of maintaining physical activity throughout life to preserve neurological health and cognitive function.

Athletes, known for their high physical performance and resilience, face significant risks when dealing with the aftermath of concussions or battling the effects of COVID-19. Concussions have been extensively documented to impair cognitive function, physical abilities, and emotional balance, factors crucial for peak athletic performance [74,75]. The symptoms of PCS—including headaches, dizziness, and cognitive difficulties—can severely impact an athlete’s ability to train, compete, and engage in daily activities [76,77].

COVID-19 adds another layer of complexity to this scenario. Beyond the acute phase of infection, the post-acute sequelae of SARS-CoV-2 infection (PASC), commonly referred to as Long COVID, encompasses a range of symptoms such as fatigue, dyspnea, and cognitive fog, mirroring some aspects of PCS [78,79,80]. These overlapping symptoms can complicate the diagnosis, management, and recovery processes for athletes, potentially extending their time away from sport and hindering performance long after the initial recovery period.

The confluence of these conditions necessitates a tailored, interdisciplinary approach to manage athlete health, emphasizing the need for comprehensive clinical evaluations and a cautious return-to-play protocol. Given the potential for prolonged recovery times and the risk of exacerbating symptoms, medical professionals should adopt a conservative strategy, prioritizing the athlete’s long-term health over immediate return to competition [81,82].

Rehabilitation programs should be designed to address both the physical and cognitive aspects of recovery, ensuring athletes receive support tailored to their specific needs. Cognitive rehabilitation, physical therapy, and gradual exercise programs can help address the symptoms of PCS and PASC, facilitating a safer return to athletic activities [83,84].

Moreover, the psychological impact of prolonged recovery, fear of reinfection, or anxiety about returning to play post-concussion should not be underestimated (Table 2). Psychological support services and counseling should be integrated into the recovery process, providing athletes with the mental health resources needed to navigate this challenging period [85,86].

The intersection of COVID-19 and concussion within athletics demands a careful, multidisciplinary approach to ensure the safe and effective return of athletes to sport. Clinicians and sports medicine professionals must remain vigilant, adopting evidence-based practices to manage these complex cases. Tailoring recovery protocols to each athlete’s unique circumstances will be paramount in safeguarding their health and ensuring their long-term success in their respective sports.

## 6. Dual Challenge of COVID-19 and Concussions

Athletes contending with the sequelae of both post-COVID-19 neurological syndrome (PCNS/PASC) and persistent post-concussive symptoms (PPCS) encounter a multifaceted challenge. This dual burden complicates the clinical picture, as both conditions independently contribute to symptoms like exercise intolerance, cognitive difficulties, and mental health issues, which are further exacerbated when combined.

Exercise Intolerance: This symptom is particularly challenging for athletes, as both PCNS/PASC and PPCS can lead to significant reductions in physical performance. The underlying causes—ranging from systemic inflammation in PCNS/PASC to autonomic dysfunction in PCS—can lead to a decreased ability to engage in high-intensity physical activities, which are fundamental in most sports (Table 1 and Table 2) [108,109].

Cognitive Difficulties: Impairments in concentration, memory, and other cognitive functions can be debilitating for athletes, whose sports often require quick decision-making, strategic thinking, and sustained attention (Table 1) [74,75]. The cognitive load of processing game dynamics in the presence of these symptoms can lead to suboptimal performance and increase the risk of further injury.

Mental Health Issues: The psychological impact of both conditions cannot be understated. Athletes, often accustomed to high levels of physical activity and competition, may experience heightened levels of anxiety, depression, or stress due to their prolonged recovery and uncertainty about their sports career (Table 1) [76,77,78].

## 7. Adapting Management Strategies

Given these challenges, sports medicine practitioners must adopt a dynamic and individualized approach to managing athletes recovering from COVID-19, concussion, or both.

Close Monitoring of Symptoms: Regular and thorough assessment of symptoms is critical (Table 2). This involves not only tracking the resolution or persistence of symptoms but also understanding how they manifest during different levels of physical activity.

Gradual Increase in Physical Activity: The cornerstone of returning to play post-injury or illness is a carefully graded exercise program. This approach helps in gauging the athlete’s tolerance to physical activity and in identifying any exacerbation of symptoms, which could indicate a need to slow down the progression.

Psychological Support: Given the mental health implications, providing psychological support is as crucial as addressing physical health. This support can range from counseling services to strategies aimed at coping with stress and anxiety related to the recovery process.

Baseline and Post-Injury Neurologic Testing: Preseason baseline testing becomes invaluable in this context, as it offers a reference point to compare an athlete’s post-injury state. Subsequent neurologic testing aids in determining the extent of recovery and readiness to resume sport-specific activities.

Return-to-Sport Decisions: Decisions regarding return to sport should be multifactorial, considering not only the resolution of symptoms but also the athlete’s overall physical and mental readiness. This decision-making process must be collaborative, involving athletes, healthcare providers, and coaches, ensuring that the athlete’s health and safety are prioritized.

The intersection of COVID-19 and concussion in athletes underscores the intricacies of managing sports-related medical conditions. It demands a holistic approach that transcends conventional treatment protocols, emphasizing the need for personalized care strategies that cater to the multifaceted needs of athletes. The ultimate goal is to facilitate a safe return to sport while safeguarding the long-term health and well-being of the athlete, a challenge that requires astute clinical acumen, interdisciplinary collaboration, and a deep understanding of the unique demands of athletic performance.

## 8. Future Directions and Conclusions

As we peer into the future of managing post-COVID-19 neurological syndrome (PCNS/PASC) and PPCS, it becomes increasingly evident that the journey is as much about unearthing new knowledge as it is about refining the current practices. This path is paved with both challenges and opportunities, compelling healthcare providers to evolve continually in their understanding and management of these complex neurological conditions.

Moving forward, healthcare providers should prioritize the following actions in managing PPCS:

- Implement Multidisciplinary Care: Adopt needs-based multidisciplinary care models that integrate medical, psychological, and rehabilitative expertise to effectively address the diverse needs of PPCS patients.
- Tailor Rehabilitation Programs: Develop personalized rehabilitation programs guided by pathophysiological insights and comprehensive assessment to target specific symptoms and underlying mechanisms contributing to PPCS.
- Utilize Technology: Harness technology for treatment delivery and monitoring to enhance precision in symptom management and rehabilitation progress tracking, as recommended.
- Emphasize BioPsychoSocial Assessment: Ensure accurate diagnosis and treatment planning through a BioPsychoSocial framework, addressing biological, psychological, and social factors that influence PPCS outcomes.
- Promote Further Research: Support ongoing research efforts to refine diagnostic criteria, optimize rehabilitation techniques, and improve long-term outcomes for PPCS patients.

## 9. Research Gaps: Uncharted Territories in Neurological Effects

In the current research landscape, there is a distinct lack of comprehensive longitudinal studies addressing the enduring neurological implications of COVID-19 and concussion. The collective response and understanding of these conditions have been largely reactionary, focusing primarily on immediate clinical needs. For a more complete understanding, it is imperative to transition towards proactive and thorough research initiatives. A critical area of focus should be the longitudinal impact, particularly examining the long-term cognitive, psychological, and physical effects of post-COVID-19 neurological syndrome (PCNS)/post-acute sequelae of SARS-CoV-2 infection (PASC) and PPCS. Understanding these long-term effects is vital for developing effective management strategies. Another pivotal aspect is the optimization of treatment strategies. There is a pressing need to devise and refine therapeutic interventions that are specifically tailored to the unique characteristics of each condition. This involves a nuanced approach that takes into account the individual variances of each condition and how they manifest in different patients. Equally important is the development of rehabilitation protocols. These protocols should be dynamic, capable of adapting to the individual needs of patients, and should evolve in tandem with the growing understanding of these neurological conditions. The aim is to provide rehabilitation that is as personalized and effective as possible. Lastly, the identification of predictive outcomes is crucial. Recognizing early markers or predictors of long-term outcomes can play a significant role in guiding early intervention strategies. This proactive approach could significantly improve the management of these conditions by allowing for earlier, more targeted interventions, potentially altering the course of recovery and long-term impact. Overall, by delving into these areas, we can hope to gain a more comprehensive understanding of the long-term effects of COVID-19 and concussion, leading to more effective treatment and management strategies for those affected.

## 10. Holistic Approach: The Convergence of Disciplines

The complexity of post-COVID neurological syndrome (PCNS), post-acute sequelae of SARS-CoV-2 infection (PASC), and post-Post-COVID Syndrome (PPCS) demands a treatment paradigm that extends beyond conventional approaches. Embracing a holistic, interdisciplinary method is not only advantageous but indispensable for effective management of these conditions. This comprehensive approach orchestrates expertise from diverse fields such as neurology, psychiatry, physical therapy, sports, and occupational medicine. Each of these disciplines contributes its distinct perspective and specialized skills, enriching the treatment landscape and the well-being of the athlete [110,111], supporting them in increasing personal achievements to constantly obtain remarkable performances in sports, but in at the same time it protects their occupational health in the conditions of extreme effort characteristic of competitive activity [112].

In this collaborative framework, comprehensive care strategies emerge as a cornerstone. This involves the integration of various therapeutic modalities, ensuring a holistic consideration of all aspects of a patient’s health. The treatment is not one-size-fits-all; rather, it is finely tuned to the individual’s needs. Personalized treatment plans are crafted with meticulous attention to the unique clinical manifestations of each patient, ensuring that interventions are as individualized as the patients themselves. Moreover, this collaborative ethos fosters the development of innovative rehabilitation techniques. By pooling the collective expertise of these varied disciplines, novel rehabilitation strategies are conceived. These strategies are not only more effective but are also centered around the patient’s specific needs and conditions. Such innovation is crucial in addressing the multifaceted challenges presented by PCNS, PASC, and PPCS, ensuring a patient-centered approach that is both cutting-edge and deeply responsive to individual health trajectories.

## 11. Final Remarks

The journey through the realms of PCNS/PASC and PPCS is a testament to the dynamic nature of medical science. The parallels and distinctions between these conditions provide a rich tapestry of clinical insights, each representing a unique challenge or opportunity. As understanding deepens, strategies in treatment and rehabilitation must adapt, characterized by a blend of vigilance, innovation, and research.

In conclusion, the task at hand for healthcare providers is twofold. Firstly, to continuously expand the knowledge base through dedicated research and study, filling the gaps that currently exist in understanding these conditions. Secondly, to apply this evolving knowledge in a manner that is holistic, patient-centered, and interdisciplinary, ensuring that approaches to treatment are as multifaceted as the conditions being managed. In doing so, outcomes for patients affected by PCNS/PASC and PPCS can be improved, while also paving the way for future advancements in the field of neurological care.

## Figures and Tables

**Table 1:** Comparative analysis of PCNS/PASC and PCS.

Aspect	PCNS/PASC	PCS
Symptoms	Cognitive difficulties (brain fog), headaches, dizziness, fatigue, sleep disturbances, mood changes, anosmia, ageusia	Cognitive difficulties (memory, attention), headaches, dizziness, fatigue, sleep disturbances, mood changes, anosmia, ageusia
Pathophysiology	- Involvement of ACE2 receptors in neuronal cells - Systemic immune response and inflammation - Endothelial dysfunction and BBB disruption from cytokine release - Specific impacts on olfactory and taste pathways, autonomic regulation	- Neuronal depolarization, altered glucose metabolism - Release of excitatory neurotransmitters - Ionic shifts (potassium efflux, sodium/calcium influx) - Energy crisis and mitochondrial dysfunction - Axonal damage, neuroinflammation
Mechanisms of BBB Disruption	Systemic inflammation and cytokine release from immune response to SARS-CoV-2	Mechanical forces from initial injury causing ionic fluxes and metabolic disturbances
Neurological Pathways	Olfactory and taste pathways, autonomic regulation	Cognitive processing, vestibular function, post-traumatic headache syndromes
Current Treatment Approaches	- Symptomatic relief and supportive care - Cognitive rehabilitation - Psychological support - Physical therapy - Pharmacological interventions for specific symptoms	- Symptomatic relief and supportive care - Vestibular therapy - Cognitive therapy - Psychotherapy - Pharmacological treatments for headaches, sleep disturbances, mood disorders
Role of Exercise and Rehab	- Progressive aerobic exercises - Resistance training - Tailored exercise routines	- Gradual return-to-activity protocol (stepwise approach) - Balance between rest and activity
Implications for Athletes	- Exercise intolerance from systemic inflammation - Cognitive difficulties affecting performance - Psychological impact of prolonged recovery	- Exercise intolerance from autonomic dysfunction - Cognitive difficulties affecting performance - Psychological impact of prolonged recovery

ACE2: angiotensin-converting enzyme 2; BBB: blood–brain barrier; SARS-CoV-2: severe acute respiratory syndrome coronavirus 2.

**Table 2:** Rehabilitation strategies and implications for athletes.

Aspect	PCNS/PASC	PCS
Rehabilitation Goals	- Reduction of cognitive symptoms - Improvement of quality of life - Psychological support - Physical rehabilitation	- Reduction of post-concussion symptoms - Recovery of cognitive and physical functions - Psychological support
Types of Exercises and Therapies	- Progressive aerobic exercises - Resistance training - Cognitive therapy - Physical therapy	- Gradual return to activity protocol - Vestibular therapy - Cognitive therapy - Physical therapy
Pharmacological Interventions	- Treatment of headaches - Treatment of neuropathic pain - Medications for sleep disorders	- Treatment of persistent headaches - Medications for sleep disorders - Antidepressants and anxiolytics
Psychological Support	- Counseling and psychological support - Management of anxiety and depression	- Cognitive behavioral therapy - Stress and anxiety management
Implications for Athletes	- Exercise intolerance due to systemic inflammation - Cognitive difficulties affecting performance - Psychological impact of prolonged recovery	- Exercise intolerance due to autonomic dysfunction - Cognitive difficulties affecting performance - Psychological impact of prolonged recovery
Return to Sport Activity	- Cautious and gradual strategy - Close monitoring of symptoms - Personalized exercise program	- Gradual and controlled return to activity protocol - Close monitoring of symptoms - Balance between rest and activity
Factors to Consider	- Severity of PCNS/PASC symptoms - Impact on cognitive and physical functions - Need for continuous support	- Severity of PCS symptoms - Impact on cognitive and physical functions - Need for continuous support

PCNS: post-COVID-19 neurological syndrome; PASC: post-acute sequelae of SARS-CoV-2 infection; PCS: post-COVID-19 syndrome.

## References

[1] Winter D., Braw Y. (2022). COVID-19: Impact of diagnosis threat and suggestibility on subjective cognitive complaints. Int. J. Clin. Health Psychol..

[2] Wang D.H., Trojian T.H., Leddy J.J. (2022). Post–COVID-19 Neurological Syndrome and Concussion. Clin. J. Sport Med..

[3] Pierpoint L.A., Collins C.L. (2021). Epidemiology of Sport-Related Concussion. Clin. Sports Med..

[4] Harmon K.G., Clugston J.R., Dec K., Hainline B., Herring S., Kane S.F., Kontos A.P., Leddy J.J., McCrea M., Poddar S.K. (2019). American Medical Society for Sports Medicine position statement on concussion in sport. Br. J. Sports Med..

[5] Rosenthal J.A., Foraker R.E., Collins C.L., Comstock R.D. (2014). National High School Athlete Concussion Rates From 2005–2006 to 2011–2012. Am. J. Sports Med..

[6] Bakhos L.L., Lockhart G.R., Myers R., Linakis J.G. (2010). Emergency department visits for concussion in young child athletes. Pediatrics.

[7] Iverson G.L., Wojtowicz M., Brooks B.L., Maxwell B.A., Atkins J.E., Zafonte R., Berkner P.D. (2020). High School Athletes With ADHD and Learning Difficulties Have a Greater Lifetime Concussion History. J. Atten. Disord..

[8] Kuhn A.W., Solomon G.S. (2015). Concussion in the National Hockey League: A systematic review of the literature. Concussion.

[9] Raukar N.P., Cooper L.T. (2021). Implications of SARS-CoV-2-Associated Myocarditis in the Medical Evaluation of Athletes. Sports Health.

[10] Symanski J.D., Tso J.V., Phelan D.M., Kim J.H. (2022). Myocarditis in the Athlete: A Focus on COVID-19 Sequelae. Clin. Sports Med..

[11] Kim J.H., Levine B.D., Phelan D., Emery M.S., Martinez M.W., Chung E.H., Thompson P.D., Baggish A.L. (2021). Coronavirus Disease 2019 and the Athletic Heart: Emerging Perspectives on Pathology, Risks, and Return to Play. JAMA Cardiol..

[12] Daniels C.J., Rajpal S., Greenshields J.T., Rosenthal G.L., Chung E.H., Terrin M., Jeudy J., Mattson S.E., Law I.H., Borchers J. (2021). Prevalence of Clinical and Subclinical Myocarditis in Competitive Athletes With Recent SARS-CoV-2 Infection: Results From the Big Ten COVID-19 Cardiac Registry. JAMA Cardiol..

[13] Groppa S.A., Ciolac D., Duarte C., Garcia C., Gasnaș D., Leahu P., Efremova D., Gasnaș A., Bălănuță T., Mîrzac D. (2022). Molecular Mechanisms of SARS-CoV-2/COVID-19 Pathogenicity on the Central Nervous System: Bridging Experimental Probes to Clinical Evidence and Therapeutic Interventions. Adv. Exp. Med. Biol..

[14] Najjar S., Najjar A., Chong D.J., Pramanik B.K., Kirsch C., Kuzniecky R.I., Pacia S.V., Azhar S. (2020). Central nervous system complications associated with SARS-CoV-2 infection: Integrative concepts of pathophysiology and case reports. J Neuroinflammation.

[15] Spudich S., Nath A. (2022). Nervous system consequences of COVID-19. Science.

[16] Berger J.R. (2020). COVID-19 and the nervous system. J. Neurovirol..

[17] Premraj L., Kannapadi N., Briggs J., Seal S., Battaglini D., Fanning J.P., Suen J.Y., Robba C., Fraser J.F., Cho S.M. (2022). Mid and Long-Term Neurological and Neuropsychiatric Manifestations of Post-COVID-19 Syndrome: A Meta-Analysis. J. Neurol. Sci..

[18] Mavroudis I., Kazis D., Chowdhury R., Petridis F., Costa V., Balmuș I.-M., Ciobîcã A., Luca A.-C., Radu I., Dobrin R. (2022). Post-Concussion Syndrome and Chronic Traumatic Encephalopathy: Narrative Review on the Neuropathology, Neuroimaging and Fluid Biomarkers. Diagnostics.

[19] Yong S.J. (2021). Long COVID or Post-COVID-19 Syndrome: Putative Pathophysiology, Risk Factors, and Treatments. Infect. Dis..

[20] Batiha G.E., Al-Kuraishy H.M., Al-Gareeb A.I., Welson N.N. (2022). Pathophysiology of Post-COVID Syndromes: A New Perspective. Virol. J..

[21] Castanares-Zapatero D., Chalon P., Kohn L., Dauvrin M., Detollenaere J., De Noordhout C.M., Jong C.P.-D., Cleemput I., Van Den Heede K. (2022). Pathophysiology and Mechanism of Long COVID: A Comprehensive Review. Ann. Med..

[22] Mehandru S., Mérad M. (2022). Pathological Sequelae of Long-Haul COVID. Nat. Immunol..

[23] Barlow K.M., Brooks B.L., Esser M.J., Kirton A., Mikrogianakis A., Zemek R.L., MacMaster F.P., Nettel-Aguirre A., Yeates K.O., Kirk V. (2020). Efficacy of Melatonin in Children With Postconcussive Symptoms: A Randomized Clinical Trial. Pediatrics.

[24] Barlow K.M., Brooks B.L., MacMaster F.P., Kirton A., Seeger T., Esser M., Crawford S., Nettel-Aguirre A., Zemek R., Angelo M. (2014). A double-blind, placebo-controlled intervention trial of 3 and 10 mg sublingual melatonin for post-concussion syndrome in youths (PLAYGAME): Study protocol for a randomized controlled trial. Trials.

[25] Wongchitrat P., Shukla M., Sharma R., Govitrapong P., Reiter R.J. (2021). Role of Melatonin on Virus-Induced Neuropathogenesis—A Concomitant Therapeutic Strategy to Understand SARS-CoV-2 Infection. Antioxidants.

[26] Iyer K.K., Zalesky A., Cocchi L., Barlow K.M. (2020). Neural correlates of sleep recovery following melatonin treatment for pediatric concussion: A randomized control trial. J. Neurotrauma.

[27] Choe M. (2016). The Pathophysiology of Concussion. Curr. Pain Headache Rep..

[28] Giza C.C., Hovda D.A. (2014). The new neurometabolic cascade of concussion. Neurosurgery.

[29] Jassam Y.N., Izzy S., Whalen M., McGavern D.B., El Khoury J. (2017). Neuroimmunology of Traumatic Brain Injury: Time for a Paradigm Shift. Neuron.

[30] Yoshino A., Hovda D.A., Kawamata T., Katayama Y., Becker D.P. (1991). Dynamic changes in local cerebral glucose utilization following cerebral conclusion in rats: Evidence of a hyper- and subsequent hypometabolic state. Brain Res..

[31] Yuen T.J., Browne K.D., Iwata A., Smith D.H. (2009). Sodium channelopathy induced by mild axonal trauma worsens outcome after a repeat injury. J. Neurosci. Res..

[32] Tang-Schomer M.D., Johnson V.E., Baas P.W., Stewart W., Smith D.H. (2012). Partial interruption of axonal transport due to microtubule breakage accounts for the formation of periodic varicosities after traumatic axonal injury. Exp. Neurol..

[33] Choe M.C., Babikian T., DiFiori J., Hovda D.A., Giza C.C. (2012). A pediatric perspective on concussion pathophysiology. Curr. Opin. Pediatr..

[34] Shenton M.E., Hamoda H.M., Schneiderman J.S., Bouix S., Pasternak O., Rathi Y., Vu M.-A., Purohit M.P., Helmer K., Koerte I. (2012). A review of magnetic resonance imaging and diffusion tensor imaging findings in mild traumatic brain injury. Brain Imaging Behav..

[35] Koerte I.K., Hufschmidt J., Muehlmann M., Lin A.P., Shenton M.E., Laskowitz D., Grant G. (2016). Advanced Neuroimaging of Mild Traumatic Brain Injury. Translational Research in Traumatic Brain Injury.

[36] Jurick S.M., Bangen K.J., Evangelista N.D., Sanderson-Cimino M., Delano-Wood L., Jak A.J. (2016). Advanced neuroimaging to quantify myelin in vivo: Application to mild TBI. Brain Inj..

[37] Li H.H., Lee S.M., Cai Y., Sutton R.L., Hovda D.A. (2004). Differential gene expression in hippocampus following experimental brain trauma reveals distinct features of moderate and severe injuries. J Neurotrauma.

[38] Shultz S.R., MacFabe D.F., Foley K.A., Taylor R., Cain D.P. (2012). Sub-concussive brain injury in the Long-Evans rat induces acute neuroinflammation in the absence of behavioral impairments. Behav. Brain Res..

[39] Chang L., Munsaka S.M., Kraft-Terry S., Ernst T. (2013). Magnetic resonance spectroscopy to assess neuroinflammation and neuropathic pain. J. Neuroimmune Pharmacol..

[40] Albrecht D.S., Granziera C., Hooker J.M., Loggia M.L. (2016). In Vivo Imaging of Human Neuroinflammation. ACS Chem. Neurosci..

[41] Henry L.C., Tremblay S., Boulanger Y., Ellemberg D., Lassonde M. (2010). Neurometabolic Changes in the Acute Phase after Sports Concussions Correlate with Symptom Severity. J. Neurotrauma.

[42] Lin A.P., Liao H.J., Merugumala S.K., Prabhu S.P., Meehan W.P., Ross B.D. (2012). Metabolic imaging of mild traumatic brain injury. Brain Imaging Behav..

[43] Alosco M.L., Jarnagin J., Rowland B., Liao H., Stern R.A., Lin A. (2017). Magnetic Resonance Spectroscopy as a Biomarker for Chronic Traumatic Encephalopathy. Semin. Neurol..

[44] Lin A.P., Ramadan S., Stern R.A., Box H.C., Nowinski C.J., Ross B.D., Mountford C.E. (2015). Changes in the neurochemistry of athletes with repetitive brain trauma: Preliminary results using localized correlated spectroscopy. Alzheimers Res. Ther..

[45] Ross B.D., Ernst T., Kreis R., Haseler L.J., Bayer S., Danielsen E., Blüml S., Shonk T., Mandigo J.C., Caton W. (1998). 1H MRS in acute traumatic brain injury. J. Magn. Reson. Imaging.

[46] Vagnozzi R., Signoretti S., Cristofori L., Alessandrini F., Floris R., Isgrò E., Ria A., Marziale S., Zoccatelli G., Tavazzi B. (2010). Assessment of metabolic brain damage and recovery following mild traumatic brain injury: A multicentre, proton magnetic resonance spectroscopic study in concussed patients. Brain.

[47] Vagnozzi R., Signoretti S., Tavazzi B., Floris R., Ludovici A., Marziali S., Tarascio G., Amorini A.M., Di Pietro V., Delfini R. (2008). Temporal window of metabolic brain vulnerability to concussion: A pilot 1H-magnetic resonance spectroscopic study in concussed athletes—Part III. Neurosurgery.

[48] Poole V.N., Abbas K., Shenk T.E., Breedlove E.L., Breedlove K.M., Robinson M.E., Leverenz L.J., Nauman E.A., Talavage T.M., Dydak U. (2014). MR spectroscopic evidence of brain injury in the non-diagnosed collision sport athlete. Dev. Neuropsychol..

[49] Braun M., Vaibhav K., Saad N.M., Fatima S., Vender J.R., Baban B., Hoda N., Dhandapani K.M. (2017). White matter damage after traumatic brain injury: A role for damage associated molecular patterns. Biochim. Biophys Acta.

[50] Foell D., Wittkowski H., Roth J. (2007). Mechanisms of disease: A “DAMP” view of inflammatory arthritis. Nat. Clin. Pract. Rheumatol..

[51] Russo M.V., McGavern D.B. (2015). Immune Surveillance of the CNS following Infection and Injury. Trends Immunol..

[52] Vénéreau E., Ceriotti C., Bianchi M.E. (2015). DAMPs from Cell Death to New Life. Front. Immunol..

[53] Yang S., Xu L., Yang T., Wang F. (2014). High-mobility group box-1 and its role in angiogenesis. J. Leukoc. Biol..

[54] van Beijnum J.R., Nowak-Sliwinska P., van den Boezem E., Hautvast P., Buurman W.A., Griffioen A.W. (2013). Tumor angiogenesis is enforced by autocrine regulation of high-mobility group box 1. Oncogene.

[55] Mitola S., Belleri M., Urbinati C., Coltrini D., Sparatore B., Pedrazzi M., Melloni E., Presta M. (2006). Cutting edge: Extracellular high mobility group box-1 protein is a proangiogenic cytokine. J. Immunol..

[56] Davalos D., Grutzendler J., Yang G., Kim J.V., Zuo Y., Jung S., Littman D.R., Dustin M.L., Gan W.-B. (2005). ATP mediates rapid microglial response to local brain injury in vivo. Nat. Neurosci..

[57] Fourgeaud L., Través P.G., Tufail Y., Leal-Bailey H., Lew E.D., Burrola P.G., Callaway P., Zagórska A., Rothlin C.V., Nimmerjahn A. (2016). TAM receptors regulate multiple features of microglial physiology. Nature.

[58] Madathil S.K., Wilfred B.S., Urankar S.E., Yang W., Leung L.Y., Gilsdorf J.S., Shear D.A. (2018). Early Microglial Activation Following Closed-Head Concussive Injury Is Dominated by Pro-Inflammatory M-1 Type. Front. Neurol..

[59] Cristofori L., Tavazzi B., Gambin R., Vagnozzi R., Signoretti S., Amorini A.M., Fazzina G., Lazzarino G. (2005). Biochemical analysis of the cerebrospinal fluid: Evidence for catastrophic energy failure and oxidative damage preceding brain death in severe head injury: A case report. Clin. Biochem..

[60] Mouzon B.C., Bachmeier C., Ferro A., Ojo J., Crynen G., Acker C.M., Davies P., Mullan M., Stewart W., Crawford F. (2014). Chronic neuropathological and neurobehavioral changes in a repetitive mild traumatic brain injury model. Ann. Neurol..

[61] Kierans A.S., Kirov I.I., Gonen O., Haemer G., Nisenbaum E., Babb J.S., Grossman R.I., Lui Y.W. (2014). Myoinositol and glutamate complex neurometabolite abnormality after mild traumatic brain injury. Neurology.

[62] Shutter L., Tong K.A., Holshouser B.A. (2004). Proton MRS in acute traumatic brain injury: Role for glutamate/glutamine and choline for outcome prediction. J. Neurotrauma.

[63] Ashwal S., Holshouser B., Tong K., Serna T., Osterdock R., Gross M., Kido D. (2004). Proton MR spectroscopy detected glutamate/glutamine is increased in children with traumatic brain injury. J. Neurotrauma.

[64] Purves D., Augustine G.J., Fitzpatrick D., Katz L.C., LaMantia A.-S., McNamara J.O., Williams S.M. (2001). Glutamate Receptors. Neuroscience.

[65] Guerriero R.M., Giza C.C., Rotenberg A. (2015). Glutamate and GABA imbalance following traumatic brain injury. Curr. Neurol. Neurosci. Rep..

[66] Rao V.L., Baþkaya M.K., Doðan A., Rothstein J.D., Dempsey R.J. (1998). Traumatic brain injury down-regulates glial glutamate transporter (GLT-1 and GLAST) proteins in rat brain. J. Neurochem..

[67] Zander N.E., Piehler T., Banton R., Benjamin R. (2016). Effects of repetitive low-pressure explosive blast on primary neurons and mixed cultures. J. Neurosci. Res..

[68] Paiva W.S., De Andrade A.F., Monaco, Amorim R.L.O., Tavares W.M., DE Figueiredo E.G., Teixeira M.J. (2011). Serum sodium disorders in patients with traumatic brain injury. Ther. Clin. Risk Manag..

[69] Grover H., Qian Y., Boada F.E., Lakshmanan K., Flanagan S., Lui Y.W. (2018). MRI Evidence of Altered Callosal Sodium in Mild Traumatic Brain Injury. AJNR Am. J. Neuroradiol..

[70] Dwyer B., Katz D.I. (2018). Postconcussion Syndrome. Handbook of Clinical Neurology.

[71] Mavroudis I., Ciobîcã A., Luca A.-C., Balmuș I.-M. (2023). Post-Traumatic Headache: A Review of Prevalence, Clinical Features, Risk Factors, and Treatment Strategies. J. Clin. Med..

[72] Smulligan K.L., Wingerson M.J., Seehusen C.N., Wilson J.C., Howell D.R. (2022). Postconcussion Dizziness Severity Predicts Daily Step Count during Recovery among Adolescent Athletes. Med. Sci. Sports Exerc..

[73] Manley G., Gardner A.J., Schneider K., Guskiewicz K.M., Bailes J.E., Cantu R.C., Castellani R.J., Turner M.S., Jordan B., Randolph C. (2017). A Systematic Review of Potential Long-Term Effects of Sport-Related Concussion. Br. J. Sports Med..

[74] Ceban F., Ling S., Lui L.M.W., Lee Y., Gill H., Teopiz K.M., Rodrigues N.B., Subramaniapillai M., Di Vincenzo J.D., Cao B. (2022). Fatigue and Cognitive Impairment in Post-COVID-19 Syndrome: A Systematic Review and Meta-Analysis. Brain Behav. Immun..

[75] McInnes K., Friesen C., MacKenzie D., Westwood D.A., Boe S.G. (2017). Mild Traumatic Brain Injury (mTBI) and Chronic Cognitive Impairment: A Scoping Review. PLoS ONE.

[76] Brustman K., Eagle S.R., Mucha A., Trbovich A., Collins M.W., Kontos A.P. (2020). Association of Sleep Symptoms with Mood and Vestibular Subtypes Following Sport-Related Concussion. Appl. Neuropsychol. Child..

[77] Stefanou M., Palaiodimou L., Bakola E., Smyrnis N., Papadopoulou Ì., Paraskevas G.P., Rizos E., Boutati E., Grigoriadis N., Krogias C. (2022). Neurological Manifestations of Long-COVID Syndrome: A Narrative Review. Ther. Adv. Chronic Dis..

[78] Aiyegbusi O.L., Hughes S., Turner G., Rivera S.C., McMullan C., Chandan J.S., Haroon S., Price G., Davies E.H., Nirantharakumar K. (2021). Symptoms, Complications and Management of Long COVID: A Review. J. R. Soc. Med..

[79] Crook H., Raza S., Nowell J., Young M., Edison P. (2021). Long Covid—Mechanisms, Risk Factors, and Management. BMJ.

[80] Smulligan K.L., Wilson J.C., Seehusen C.N., Wingerson M.J., Magliato S.N., Howell D.R. (2021). Postconcussion Dizziness, Sleep Quality, and Postural Instability: A Cross-Sectional Investigation. J. Athl. Train..

[81] Ashina H., Porreca F., Anderson T., Amin F.M., Ashina M., Schytz H.W., Dodick D.W. (2019). Post-Traumatic Headache: Epidemiology and Pathophysiological Insights. Nat. Rev. Neurol..

[82] Tana C., Bentivegna E., Cho S.-J., Harriott A.M., García-Azorín D., Labastida-Ramírez A., Ornello R., Raffaelli B., Beltrán E.R., Ruscheweyh R. (2022). Long COVID Headache. J. Headache Pain.

[83] Foster E., Bayley M., Langer L., Saverino C., Chandra T., Barnard C., Comper P. (2022). The Toronto Concussion Study: Sense of Smell Is Not Associated with Concussion Severity or Recovery. Brain Inj..

[84] Najafloo R., Majidi J., Asghari A., Aleemardani M., Kamrava S.K., Simorgh S., Seifalian A., Bagher Z., Seifalian A.M. (2021). Mechanism of Anosmia Caused by Symptoms of COVID-19 and Emerging Treatments. ACS Chem. Neurosci..

[85] Yoen H., Yoo R.E., Choi S.H., Kim E., Oh B.M., Yang D., Hwang I.-K., Kang K.M., Yun T.J., Kim J.H. (2021). Blood-Brain Barrier Disruption in Mild Traumatic Brain Injury Patients with Post-Concussion Syndrome: Evaluation with Region-Based Quantification of Dynamic Contrast-Enhanced MR Imaging Parameters Using Automatic Whole-Brain Segmentation. Korean J. Radiol..

[86] Kempuraj D., Aenlle K., Cohen J.R., Mathew A.S., Isler D., Pangeni R.P., Nathanson L., Theoharides T.C., Klimas N.G. (2023). COVID-19 and Long COVID: Disruption of the Neurovascular Unit, Blood-Brain Barrier, and Tight Junctions. Neuroscientist.

[87] Raj S.R., Arnold A.C., Barboi A., Claydon V.E., Limberg J.K., Lucci V.-E.M., Numan M.T., Peltier A., Snapper H., Vernino S. (2021). Long-COVID Postural Tachycardia Syndrome: An American Autonomic Society Statement. Clin. Auton. Res..

[88] Skjeldal O.H., Skandsen T., Kinge E., Glott T., Solbakk A. (2022). Long-Term Post-Concussion Symptoms. Tidsskr. Nor. Laegeforening.

[89] Davis H., McCorkell L., Vogel J.M., Topol E.J. (2023). Long COVID: Major Findings, Mechanisms and Recommendations. Nat. Rev. Microbiol..

[90] Chee Y.J., Fan B.E., Young B.E., Dalan R., Lye D.C. (2022). Clinical Trials on the Pharmacological Treatment of Long COVID: A Systematic Review. J. Med. Virol..

[91] Quatman-Yates C., Hunter-Giordano A., Shimamura K.K., Landel R.F., Alsalaheen B., Hanke T., McCulloch K., Altman R.D., Beattie P., Berz K. (2020). Physical Therapy Evaluation and Treatment after Concussion/Mild Traumatic Brain Injury. J. Orthop. Sports Phys. Ther..

[92] Komici K., Bianco A., Perrotta F., Dello Iacono A., Bencivenga L., D’Agnano V., Rocca A., Bianco A., Rengo G., Guerra G. (2021). Clinical Characteristics, Exercise Capacity and Pulmonary Function in Post-COVID-19 Competitive Athletes. J. Clin. Med..

[93] Singh I., Joseph P., Heerdt P.M., Cullinan M., Lutchmansingh D.D., Gulati M., Possick J.D., Systrom D.M., Waxman A.B. (2022). Persistent Exertional Intolerance After COVID-19: Insights From Invasive Cardiopulmonary Exercise Testing. Chest.

[94] Baratto C., Caravita S., Faini A., Perego G.B., Senni M., Badano L.P., Parati G. (2021). Impact of COVID-19 on exercise pathophysiology: A combined cardiopulmonary and echocardiographic exercise study. J. Appl. Physiol..

[95] Ladlow P., O’sullivan O., Bennett A.N., Barker-Davies R., Houston A., Chamley R., May S., Mills D., Dewson D., Rogers-Smith K. (2022). The effect of medium-term recovery status after COVID-19 illness on cardiopulmonary exercise capacity in a physically active adult population. J. Appl. Physiol..

[96] Richmond L.M. (2022). No Surprises Act Brings New Billing Rules, Disclosures; Doctors Sue. Psychiatr. News.

[97] Arbula S., Pisanu E., Bellavita G., Menichelli A., Lunardelli A., Furlanis G., Manganotti P., Cappa S., Rumiati R. (2024). Insights into attention and memory difficulties in post-COVID syndrome using standardized neuropsychological tests and experimental cognitive tasks. Sci. Rep..

[98] Warren S., Drake J., Wu C.K. (2022). Cognitive Complications of COVID-19 Infection. Rhode Isl. Med. J..

[99] Daroische R., Hemminghyth M.S., Eilertsen T.H., Breitve M.H., Chwiszczuk L.J. (2021). Cognitive Impairment After COVID-19-A Review on Objective Test Data. Front. Neurol..

[100] Son C., Hegde S., Smith A., Wang X., Sasangohar F. (2020). Effects of COVID-19 on College Students’ Mental Health in the United States: Interview Survey Study. J. Med. Internet Res..

[101] Wang X., Hegde S., Son C., Keller B., Smith A., Sasangohar F. (2020). Investigating Mental Health of US College Students During the COVID-19 Pandemic: Cross-Sectional Survey Study. J. Med. Internet Res..

[102] Romano R.J., Devarakonda L. (2023). Pandemic-related mental health crises should serve as wake-up call for NCAA. Coll. Athl. Law.

[103] McLellan M., Heffernan C., Xu J., Billimek J., Kim B.Y. (2022). Mental Health and Substance Use in NCAA Athletes in the Context of the COVID-19 Pandemic and Lockdown. Cureus.

[104] Zhao E., Tranovich M.J., DeAngelo R., Kontos A.P., Wright V.J. (2016). Chronic exercise preserves brain function in masters athletes when compared to sedentary counterparts. Phy. Sportsmed..

[105] Tseng B.Y., Uh J., Rossetti H.C., Cullum C.M., Diaz-Arrastia R.F., Levine B.D., Lu H., Zhang R. (2013). Masters athletes exhibit larger regional brain volume and better cognitive performance than sedentary older adults. J. Magn. Reason. Imaging.

[106] Hüttermann S., Memmert D. (2014). Does the inverted-U function disappear in expert athletes? An analysis of the attentional behavior under physical exercise of athletes and non-athletes. Physiol. Behav..

[107] Mischel N.A., Llewellyn-Smith I.J., Mueller P.J. (2014). Physical (in)activity-dependent structural plasticity in bulbospinal catecholaminergic neurons of rat rostral ventrolateral medulla. J. Comp. Neurol..

[108] Renz-Polster H., Scheibenbogen C. (2022). Post-COVID-Syndrom Mit Fatigue Und Belastungsintoleranz: Myalgische Enzephalomyelitis Bzw. Chronisches Fatigue-Syndrom. Inn. Med..

[109] Pelo R., Suttman E., Fino P.C., McFarland M., Dibble L.E., Cortez M. (2023). Autonomic Dysfunction and Exercise Intolerance in Concussion: A Scoping Review. Clin. Auton. Res..

[110] Verow P. (2006). Sports and occupational medicine: Two sides of the same coin?. Occup. Med..

[111] Naghavi S. (2007). Sports medicine concept in occupational medicine. Occup. Med..

[112] (2017). Sánchez A, Sports medicine vs occupational medicine: Two divergent specialties with a common past. Arch. Med. Deporte.

